# Are pregnancy and parity associated with telomere length? A systematic review

**DOI:** 10.1186/s12884-023-06011-8

**Published:** 2023-10-17

**Authors:** Nourit Houminer-Klepar, Shiran Bord, Elissa Epel, Orna Baron-Epel

**Affiliations:** 1 School of Public Health, Faculty of Social Welfare and Health Sciences, University of Haifa, Mount Carmel, 31905 Haifa, Israel; 2 Health Systems Management Department, The Max Stern Yezreel Valley College, 1930600 Yezreel Valley, Israel; 3 Department of Psychiatry and Behavioral Sciences, University of California, 675 18th St, San Francisco, CA 94107 USA

**Keywords:** Telomere, Telomere shortening, Parity, Pregnancy, Reproduction, Reproductive history

## Abstract

**Background:**

Women's reproduction requires increased energy demands, which consequently may lead to cellular damage and aging. Hence, Telomere Length (TL), a biomarker of biological aging and health status may possibly serve as a biomarker of reproductive effort. The aim of this systematic review is to evaluate telomere dynamics throughout pregnancy and the association between parity and TL.

**Methods:**

A systematic search was conducted across seven databases including CINAHL, Cochrane, PsycINFO, Proquest, PubMed; Scopus; and Web of Science, using keywords and MeSH descriptors of parity and TL. Predefined inclusion and exclusion criteria were used to screen abstracts and titles. After the removal of duplicates, 3431 articles were included in the primary screening, narrowed to 194 articles included in the full-text screening. Consensus was reached for the 14 studies that were included in the final review, and the Newcastle–Ottawa scale (NOS) was utilized to assess the quality of the selected studies. A mini meta-analysis utilized JASP 0.17.3 software and included 4 applicable studies, comprising a total of 2564 participants to quantitatively assess the estimated effect size of parity on TL.

**Results:**

Of the 11 studies reviewed on parity and TL, four demonstrated a negative correlation; one – a positive correlation and six -found no correlation. Studies demonstrating a negative correlation encompassed rigorous methodological practices possibly suggesting having more children is associated with enhanced telomere attrition. Of the four longitudinal studies assessing telomere dynamics throughout pregnancy, most found no change in TL from early pregnancy to postpartum suggesting pregnancy does not affect TL from early pregnancy to early postpartum. The meta-analysis revealed a negative, yet, non-significant effect, of the estimated effect size of parity on TL(ES = -0.009, *p* = 0.126, CI -0.021, 0.03).

**Conclusions:**

Studies assessing pregnancy, parity and TL yielded mixed results, most likely due to the different research methods utilized in each study. Improvements in study design to better understand the short-term effects of pregnancy on TL and the effect of parity on TL over time, include precise definitions of parity, comparisons of different age groups, inclusion of reproductive lifespan and statistically adjusting for potential confounders in the parity and TL relationship.

**Supplementary Information:**

The online version contains supplementary material available at 10.1186/s12884-023-06011-8.

## Background

Reproduction requires a high energy investment by women due to increased metabolic demands, as internal resources are allocated to reproductive effort [1]. This may interrupt cellular maintenance mechanisms of repair and regeneration and, over time, may lead to cellular damage and aging [2]. The Disposable Soma Theory hypothesizes that somatic maintenance is compromised with an increased number of births since physical resources are allocated to reproduction and childrearing and are not available for needed endogenous repair [3]. Even though this theory’s pertinence to humans has varied [4], previous research has suggested that women who have had more pregnancies age faster [5]. Further investigation is required since enhanced aging serves as a risk factor for chronic diseases and disorders as well as reduced longevity [6].

Telomeres, the protective "caps" at the end of chromosomes, serve as one type of biomarker for cellular aging, specifically related to replicative senescence [7]. Telomere length (TL) varies in different cell types; however, leukocyte TL is a biomarker of biological age and health status [8] and genomic instability [9] TL is also an integrated measure of allostatic load and environmental exposures as it is affected by factors such as stress, smoking, and pollution [10, 11].

Telomere shortening is a normal cellular process and while it has been associated with morbidity and mortality from psychological and physiological stressors and disorders [2, 12–14] it is important to note that TL research is a relatively new field of study, and additional research is needed to uncover the role of telomeres in human health. Current knowledge unfolds that differences in TL can be attributed to both the rate of telomere shortening over time and variations in TL at birth [15]. Adding to that, TL research confronts challenges due to variations in TL measurement methods [16] making it difficult to compare results across studies. Moreover, TL has been found to be influenced by various lifestyle factors such as inadequate nutrition [17, 18], sleep [19], physical activity [20] and obesity [21]. Since accelerated telomere shortening is associated with accelerated aging, TL may serve as a biomarker of reproductive effort [22].

Studies investigating the effects of pregnancy on women’s health, longevity, and TL have shown that physiologically, normal pregnancies are accompanied by elevated inflammation [23], dyslipidemia [24], insulin resistance and enhanced oxidative stress [25–28]. Pregnancy has been depicted as a model for aging [29] due to the similarities between aging and pregnancy’s physiological and cellular processes. Nonetheless, although pregnancy has the potential to lead to cellular damage and enhanced aging, it has a restoring effect as well, and damage is usually reversed during the postpartum period, wherein women recover back to their pre-pregnancy state with increased bodily defense mechanisms against cellular damage [29]. This, in turn, suggests pregnancy, at least in the early post-partum phase, entails mechanisms of recovery and rejuvenation, feasibly suggesting its’ net protective health effect on the woman and possibly its ability to slow the pace of biological aging [30, 31]. Ross et al. [31], utilized women’s immune cell-derived epigenetic age biomarkers, which are associated with chronological age. This study followed pregnant women throughout their pregnancy and one year postpartum, measuring their epigenetic age biomarkers such as PEAA, GrimAge, DNAm PAI-1, and immune cell population epigenetic age indices, and found that the epigenetic clocks became younger over the follow-up period [31]. Our focus is whether pregnancy changes TL, across studies that have addressed this question.

Although pregnancy can potentially strain the cardiovascular system [32, 33], elevated estrogen levels throughout pregnancy are advantageous for women’s cardiovascular health. They act as an antioxidant, lowering oxidative stress [34], halting telomere shortening, and epigenetic aging [31, 35], as opposed to testosterone, dominated in men, which is not associated with suppression of oxidative damage [36]. In addition, via different pathways, estrogen directly increases telomerase activity in women, an enzyme responsible for maintenance and elongation of telomeres [37]. An increase in telomerase activity consecutively, slows telomere shortening and promotes telomere elongation [34]. While telomerase activity is essential for maintaining TL and preventing cellular aging, it’s activation has been implicated in tumorigenesis [38]. Although telomerase does not directly cause cancer [39], it can help cancer cells grow and spread. Thus, estrogen-induced telomerase activation may have beneficial effects on TL, but the potential risks should be considered.

Short-term pregnancy related physiological stress usually resolves after childbirth, yet whether there are cumulative effects of multiple pregnancies on health is not clearly known. Multiple pregnancies potentially may induce excessive physical and psychological stress affecting repair mechanisms that can impact women’s health in the long term and exacerbate the aging process [5]. Overall, studies have demonstrated that higher parous women, going through multiple pregnancies, in comparison to lower parous or nulliparous women, are at higher risk of developing chronic diseases and syndromes, such as cardiovascular disease [32, 40], coronary heart disease [41] type 2 diabetes [42, 43] and the metabolic syndrome [44]. The risk is disease specific since parity appears to have a protective effect in certain chronic diseases, such as breast cancer [45] and endometrial and ovarian cancer [46, 47].

Studies investigating the association between parity and mortality have shown inconsistent results, some studies have shown parity to be positively correlated with mortality mainly in historical cohorts; the more children women had, the higher their mortality rate [48, 49]. However, there is a nonlinear relationship in more contemporary populations, showing a U-shaped or J-shaped mortality rate, with the highest mortality rates among nulliparous women, or in some studies women with one child, and women with more than four or five children [50–54]. A recent study on the Irish longitudinal study on aging exhibited the opposite results, showing a negative relationship between parity and mortality, the more children women had the lower their mortality risk [55].

This systematic review has two goals: 1) to assess the potential changes in TL throughout pregnancy and 2) to evaluate the association between parity and TL. To our knowledge, there are yet no reviews on this in human subjects. Sudyka [56] reviewed 33 studies, mostly non-human subjects. She found that most studies in which reproduction was experimentally manipulated, enhanced telomere attrition occurred, yet overall studies assessing the relationship between reproduction and TL have shown both positive and negative correlations. Therefore, the question of whether parity promotes or represses longevity, and, in turn, TL needs further investigation.

## Methods

This Systematic Review followed the Preferred Reporting Items for Systematic reviews and Meta-Analyses scoping review extension (PRISMA-ScR). A systematic search of studies addressing the effect of pregnancy and parity on women’s TL was conducted across seven electronic databases, including: Cumulative Index to Nursing and Allied Health (CINAHL); Cochrane, PsycINFO, Proquest, PubMed; Scopus; and Web of Science. This systematic literature search was conducted on May 10, 2021, and was subsequently updated on June 15, 2022, it used general keyword terms for each concept and MeSH descriptors of the two main concepts of parity and TL, utilizing combinations of relevant search terms. The full search strategy can be viewed in Appendix 1.

The search strategy (Appendix 1 & Appendix 2) was developed by a team of researchers and a librarian and was peer-reviewed by an additional information specialist. All studies detected through the search process were exported to the Mendeley reference manager software. They were screened by the first author for duplicates via the automatic software screening for duplicates function and manual screening. Articles identified by the search strategy were screened in two stages; the first stage- titles and abstracts were screened for relevance by one researcher, and if the required inclusion criteria were unclear from the abstract, the full-text article was retrieved to determine eligibility. Second stage – all three researchers reviewed all selected articles for secondary screening, and any disagreements on selected studies were resolved by discussion. One additional article was identified via citation searching.

The articles had to meet the following inclusion criteria: research articles (observational, experimental, and case reports) published in English that measured TL as an outcome via any methodology. In addition, studies that focused only on human subjects, specifically, pregnant and postpartum women at any age in which they can experience pregnancy and childbirth. At last, during pregnancy: only longitudinal studies were included and/or studies that mentioned women’s number of children and presented its association with TL.

Articles meeting the exclusion criteria were excluded from the review. Exclusion Criteria included: TL measured in specific female tissues: placenta tissue, cord blood, endometrial tissues, oocytes, cumulus cells, umbilical cord, fallopian tubes, ovaries (this criterion was created to ensure TL measurements that were more representative of systemic changes in TL and to focus the research question on women within the context of their lives and their interactions with the physical and social environment, and not on tissues). Studies that only focused on women with adverse obstetric outcomes: ectopic pregnancy, miscarriage, pre-eclampsia, intrauterine growth restrictions, IVF, postpartum depression, endometriosis, infertility, premature ovarian failure as well as review articles or studies that only focused on fetal/newborn TL ( to avoid potential confounding effects of these outcomes on TL measurements).

**Fig. 1:**
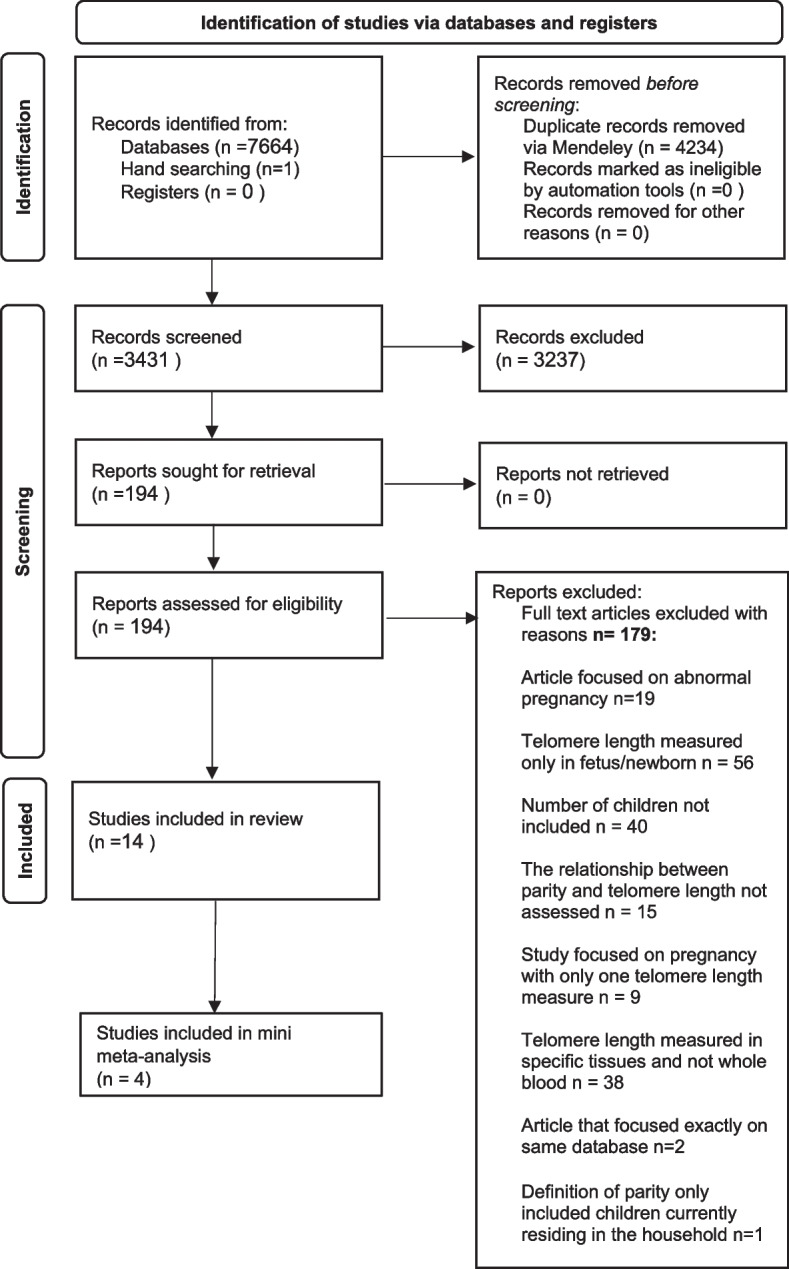
PRISMA 2020 flow diagram for new systematic reviews

We identified a total of 7664 articles through the search strategy (Fig. 1) and one additional article was identified via hand searching. After removing duplicates, 3431 articles were included in the primary screening measurements of titles and abstracts, and after removing 3237 articles, 194 articles were included in the full-text screening. No studies detected through the search of grey literature (ProQuest) met the inclusion criteria. Consensus was reached by discussion for the 14 studies that met eligibility criteria and were included in the final review (Table 1).

**Table 1:** Studies included in final review

Author/Year/Country	Title	Study Objective	Study Design/ Source of data	Sample Size/Age Range or Mean	TL method/Number of measurements	Parity defined as /Range or categories of parity	Results
**Parity and TL association as a primary measurement**							
Barha 2016 Guetamala [57]	Number of Children and TL in Women: A Prospective, Longitudinal Evaluation	Prospective Evaluation of the relationship between number of offspring and change in TL across a 13-year period	Longitudinal/Recruitment of Kaqchikel Mayan women, Guatemala	*n* = 75 29–53 (at 13 yr follow up)	Salivary and Buccal cells qPCR X2 collections: 2001- Salivary samples 2013 – Buccal epithelial cells	Total number of surviving offspring Range: 1–10 alive children	There is a positive correlation between number of children and TL. (0.059 more TL units for mothers with each additional child born between 2000–2013) (*p* = 0.045) **Positive Correlation between number of children and TL**
Kresovich 2018 US [58]	Reproductive History and Blood Telomere Length	Assess whether reproductive histories reflecting greater estrogen exposure over the life course is associated with longer blood cell telomeres	Cross Sectional/ The Sister Study, US and Peurto Rico	*n* = 1048 35–74	Whole blood qPCR X1	Number of children/Categories 4 or more and 0–1 children	Increased parity correlated with shorter rTL (β = ‐ 0.016, 95% CI: ‐0.03, 0.00, *p* = 0.07). Correlation with parity strongest for women with 4 + births relative to women with 0 or 1 births (β = -0.08, 95% CI = -0.17, 0.01, *P* = 0.07) **Negative Correlation between number of children and TL**
Lane Cordova 2017 US [59]	Gravidity is not associated with TL in a biracial cohort of middle-aged women: The Coronary Artery Risk Development in Young Adults (CARDIA) study	Assess whether there is a correlation between number of pregnancies (gravidity) and mid-life telomere length in women	Longitudinal/The Coronary Artery Risk Development in Young Adults (CARDIA) study	*n* = 620 38–45	PBMC qPCR X1	Number of times a woman has experienced a pregnancy lasting > 20 weeks/categories: 0 1 2–3 ≥ 4 pregnancies	Mean TL did not vary between women with different numbers of pregnancies (0, 1, 2–3 or 4 pregnancies (*p* = 0.51) **No Correlation between number of pregnancies and TL**
Pollack 2018 US [60]	Parity associated with TL among US reproductive age women	examine the association between parity and leukocyte telomere length	Cross-Sectional/NHANES Survey (only data between 1999–2002 included TL measure-ments)	*n* = 1554 20–44	Whole Blood qPCR X1	Number of pregnancies that resulted in live birth/Categories: 0 1 2 3 4 ≥ 5 live births	Parous women had a shorter adjusted mean leukocyte T/S ratio 4.2% (95% CI: 0.9, 7.3) compared to nulliparous women **Negative Correlation between number of live births and TL when comparing nulliparous and parous women**
Ryan 2018 Philippines [61]	Reproduction predicts shorter telomere and epigenetic age acceleration among young adult women	Assess gravidity in relation to telomere length and DNA methylation age	Longitudinal Cebu / Longitudinal Health and Nutrition Survey (CLHNS),	*n* = 821 TL *n* = 397 DNAmAge/20–22	Whole Blood qPCR X2	Number of pregnancies including stillbirths, miscarriages and live births, but not current pregnancies/Categories: 1 2 3 4 5 pregnancies	TL decreased (*p* = 0.031) and DNAmAge increased (*p* = 0.007) with gravidity, a relationship that was not dependent upon resource availability **Negative Correlation**
Michaeli 2022 Israel [62]	Leukocyte Telomere Length Correlates with Extended Female Fertility	1. Examine the association of telomeres with extended fertility in women 2. Examine difference in LTL between time period 1 (48 h postpartum) and time period 2 (5–6 months postpartum) 3. Compare LTL in women ages 30–35 years who are primiparous and grand-multiparous (6 or more deliveries)	Retrospective case–control study Recruitment of Orthodox women from Department of Obstetrics and Gynecology, Shaare Zedek Medical Center, Jerusalem, Israel	*n* = 60 {Extended Fertility (EF) (Cases) *n* = 30 Normal Fertility (NF) *n* = 30 (Control)} 43–48	Whole Blood Southern analysis the terminal restriction fragments (TRF) X2 in Extended Fertility group within 48 h after delivery and 5 months postpartum X1 in normal fertility group	Number of living children /Categories: 1 child 6–11 children	1.Average TRF length in extended fertility group (9350 bp) significantly longer than in the normal fertility group (8850 bp; *p*-value = 0.03). only among women with up to 8 children and non-significant difference in TL among women w/more than 9 children 2. Postpartum—no significant difference in leukocyte mean TRF length measured within 48 h after delivery and 5–6 months postpartum (*p*-value = ns; *n* = 11), 3. **No significant difference in TL between primiparous and multiparous women a**ged 30–35 (9540 ± 820 bp primiparous, 9490 ± 840 bp grand-multiparous women, *p*-value = ns **No correlation**
**Parity and TL association as a secondary measurement (Parity as Covariate)**							
Erdman 2017 Canada [63]	Mammographic density, blood telomere length and lipid peroxidation	Assessed whether mammographic density (MD) is related to blood telomere length	Cross-sectional Recruited from mammographic units in Toronto, Ontario, Canada	*n* = 342 mean 50.4 ± 7.2	Whole Blood qPCR and TRF X1	Parity Yes vs No Age at first child /Binary category	No correlations observed of blood TL with traditional breast cancer risk factors, specifically, age at menarche, **parity**, age at first child or family history of breast cancer **No correlation**
Flannagan 2016 Central America [64]	Sociodemographic correlates and family aggregation of leukocyte telomere length in adults and children from Mesoamerica	associations of LTL with sociodemographic and anthropometric variables and estimated LTL family aggregation in Central America	Cross-sectional Central America: Belize Costa Ri Hondura Mexico Nicaragua Panama	*n* = 174 < 30–45 > Mean: 37 ± 6.4	Whole Blood qPCR X1	Number of children/ Categories: 1 2 3 ≥ 4	In bivariate analysis, mothers’ LTL was inversely correlated with age and parity **parity was not significantly correlated** with LTL after adjustment **No Correlation**
Latour 2020 US [65]	Maternal age at last birth and leukocyte TL in a nationally representative population of peri- and postmenopausal women	Evaluate if maternal age at birth of last child is associated with leukocyte telomere length	Cross-sectional/NHANES Survey—data from1999-2002	*n* = 1232 40–85	Whole Blood qPCR X1	Number of live births/Categories 1 2 3 4 ≥ 5	A positive correlation between maternal age at last birth and LTL. suggestive evidence this association may be **restricted to those women with 1 or 2** live births (1 or 2: P-trend = 0.01**; 3 or more: *P*-trend = 0.97; *P*-interaction = 0.08;** **No Correlation**
Parks 2011 US [66]	Employment and work schedule are related to telomere length in women	Examine the association of employment and work schedule with TL and consider whether differences were related to health, behaviors and sociodemographic factors,	Cross-sectional The Sister Study	*n* = 608 35–74	Whole Blood qPCR X1	Number of children born Age at first birth /Categories 0–1 2 ≥ 3	Current schedule-related rTL and long-term full-time schedules differences were most apparent in women among other variables with three or more children (9% -13% shorter) **Negative Correlation**
**Pregnancy**							
Mitchel 2018 US [67]	Childhood adversity, social support, and telomere length among perinatal women	the association of childhood SES, childhood trauma, and current social support with TL assessed in early, mid, and late pregnancy as well as 7–11 weeks postpartum	Longitudinal	*n* = 81 18–33	PBMC qPCR X4	Number of previous births	In a linear mixed model examining changes in TL across pregnancy and postpartum visits- no significant effect of time was observed (F(3,73) = 0.12, *p* = 0.95) **No Correlation**
Zota 2019 US [10]	Association between persistent endocrine disrupting chemicals and biomarkers of inflammation and cellular aging during pregnancy and postpartum	Investigate associations between prenatal exposures to endocrine disrupting chemicals with repeated biomarker measurements of inflammation and cellular aging in women during pregnancy and the postpartum period	Longitudinal Maternal Adiposity, Metabolism, and Stress Study (MAMAs)	*n* = 103 18–42 low income overweight or obese	Whole Blood qPCR X3 At 16 weeks Pregnancy At 3 and 9 months postpartum	Parity (0 or ≥ 1)/ Binary Categoy	LTL similar across levels of covariates (parity = 0 or ≥ 1) And Biomarker measurement of TL did not differ overtime (2^nd^, 3rd trimester and 9months postpartum (*p* = 0.797) **No Correlation** **Parity/Pregnancy**
Saberi 2019 Canada [68]	Dynamics of leukocyte telomere length in pregnant women living with HIV, and HIV negative pregnant women: A longitudinal observational study	Examine longitudinal dynamics of LTL during pregnancy in a unique cohort of women living with HIV (WLWH) treated with combination antiretroviral therapy (cART), and HIV-negative control women	Longitudinal Pregnancy cohort and the Children and Women: Antiretrovirals and Markers of Aging (CARMA) cohort	*n* = 64 HIV + *n* = 41 HIV- 17–41	Whole Blood qPCR X3 during pregnancy at 13–23, > 23–30, > 30–40 weeks (for WLWH only-at 6 weeks post-partum)	Not collected	Longitudinally, LTL was similar in both groups and increased with gestation, this was more evident in women younger than 35 years **Positive correlation pregnancy**
Panelli 2022 US [69]	Leukocyte telomere dynamics across gestation in uncomplicated pregnancies and associations with stress	Assess the effect of uncomplicated pregnancies on maternal LTL	Longitudinal Recruited from March of Dimes Prematurity Research Center at Stanford University	*n* = 46 total ***n* = 32** w samples from both **time point 1 and time point 3** Mean: 30.1 ± 3.9	PBMC qPCR X3 time points Pregnancy and postpartum at < 20, 20–36, 37 – 9 weeks postpartum	Not collected (Nulliparous pregnant subjects)	There were no significant differences in LTL between Timepoints 1 and 2 (LTL T/S change − 0.03 ± 0.26, *p* = 0.39); 2 and 3 (− 0.07 ± 0.29, *p* = 0.38) or **Timepoints 1 and 3 (− 0.07 ± 0.21, *p* = 0.06)** **No Correlation**

The Newcastle–Ottawa scale (NOS), was used to assess the quality of the included studies [70]. NOS checklist for cohort studies, case–control studies and adapted for cross-sectional studies were all utilized and matched for the study type scored. The NOS scale, a star scoring system, focuses on three components: the selection, comparability, and outcome/exposure of the studies, and, in accordance with the NOS guidelines, is graded with up to five stars, two stars and three stars for each component, respectively. The quality assessment was examined independently by two authors, and any disagreements were resolved by discussion. Studies with NOS scores of 0–3, 4–6 and 7–10 were considered as low, moderate and high quality, respectively (Table 2).

**Table 2:** Quality assessment of studies included in this systematic review according to the Newcastle Ottawa scale for Cohort, Case**-**Control and Cross Sectional Studies^a^

**Cohort studies**	Representativeness of the exposed cohort	Sample Size	Response Rate	Ascertainment of Exposure	Study Controls for Relevant Confounders (Age, SES, Health)	Study Controls for Additional Factors (i.e. Stress)	Assessment of outcome	Was follow-up long enough for outcomes to occur (> 9 months)	Adequacy of follow up of cohorts (loss-to-follow-up < 20%)	**Total score**
Barha et al. 2018 [57]	**-**	*	**-**	*	*	**-**	*	*	*	6
Mitchel et al. 2018 [67]	*	*	**-**	**	*	*	*	*	**-**	8
Panelli et al. 2022 [69]	*	**-**	**-**	**	**-**	**-**	*	*	*	6
Saberi et al. 2019 [68]	**-**	*	*	**	**-**	**-**	*	*	*	7
Zota et al. 2019 [10]	**-**	*	**-**	**	*	**-**	*	*	*	7
**Case-control studies**	Adequate case definition	Representativeness of the cases	Selection of Controls	Definition of Controls	Study controls for most important factor	Study controls for an additional factor	Ascertainment of exposure	The same method of ascertainment for cases and controls	The same non-response rate for both groups	**Total score**
Michaeli et al. 2022 [62]	*	**-**	*	*	**-**	**-**	*	*	**-**	5
**Cross-sectional studies**	Representativeness of the sample	Sample size	Response rate	Ascertainment of the exposure	Study Controls for Relevant Confounders (Age, SES, Health)	Study Controls for Additional Factors (i.e. Stress)	Assessment of the outcome	Statistical test		**Total score**
Erdman et al. 2017 [63]	*	**-**	**-**	**	**-**	**-**	**	*		6
Flannagan et al. 2016 [64]	*	**-**	**-**	**	*	**-**	**	*		7
Kresovich et al. 2018 [58]	*	**-**	*	**	**-**	*	**	*		8
Latour et al. 2020 [65]	*	*	*	**	*		**	*		9
Lane Cordova et al. 2017 [59]	*	*	*	*	*	**-**	**	*		8
Parks et al. 2011 [66]	**-**	**-**	*	**	*	*	**	*		8
Pollack et al. 2018 [60]	*	*	*	**	*	**-**	**	*		9
Ryan et al. 2018 [61]	**-**	*	**-**	*	*	**-**	**	*		6

* = 1 score point in the Newcastle-Ottowa Scale

^a^Wells GA, Shea B, O’Connell D, Peterson J, Welch V, Losos M, et al. The Newcastle**-**Ottawa Scale (NOS) for assessing the quality of nonrandomized studies in meta-analyses. Oxford; 2000

At last, a mini meta-analysis utilized JASP 0.17.3 software and included 4 applicable studies, comprising a total of 2564 participants to quantitatively assess the estimated effect size of parity on TL. A fixed effects model was deemed appropriate based on the study of heterogeneity and was indicated by *p*-value 0.095; Q = 6.361 using data on effect sizes (ES) and standard error of effect sizes (SE).

## Results

All studies, included in the final review, that met eligibility criteria were published between the years 2011–2022. Eight of these studies were conducted in the United States [10, 58–60, 65–67, 69], two in Canada [63, 68], two in Central America [57, 64] one in the Philippines [61] and one in Israel [62]. Of the studies assessing parity and TL, three utilized a longitudinal research design [10, 57, 61], seven reported data from a cross-sectional study design [58–60, 63–66] and one study utilized a case control retrospective study design [62]. All studies assessing TL dynamics throughout pregnancy utilized a longitudinal research design [10, 67–69]. Overall, studies focused either on pregnant women [10, 67–69] or postpartum women [57–66].

The age range of participants in studies assessing parity and TL was 20–85, and in studies assessing TL dynamics throughout pregnancy was 17–42. Studies assessing telomere dynamics throughout pregnancy concordantly included a narrower range of ages with premenopausal women [10, 67–69]. Studies assessing parity and TL mostly comprised of a broader range of ages including a mixture of both premenopausal and postmenopausal women [57, 58, 63–66], yet some studies included a narrower range of ages of younger premenopausal women [10, 59–62]. Study sample sizes of studies assessing TL dynamics throughout pregnancy ranged between 32 to 105, with a total of 321 women, and studies assessing TL and parity had larger sample sizes ranging between 60–1554, with a total of 6534 women. All studies utilized qPCR method for TL testing with whole blood samples, except for one study that used saliva and buccal cells [57] and one study that used terminal restriction fragments (TRF) analysis [62].

The studies evaluating the relationship between parity and TL, defined parity differently, some defining parity as the number of children [58, 64], number of living children [62] and one study defined parity as the number of surviving offspring at the time of the study [57] Other studies defined parity as number of births, which was subcategorized as—live births [60, 65, 66] and number of previous births [67]. Parity was also defined as any pregnancy lasting > 20 weeks [59] and Ryan et al. (2018) in addition to live births also included pregnancies that ended as stillbirths and miscarriages [61], and at last two studies defined parity as a dichotomous variable of either multiparity or nulliparity [10, 63].

The selected articles were divided into three categories (Table 1): 1. Studies that directly assessed the relationship between number of children and TL [57–62] 2. Studies that assessed the relationship between TL and a secondary variable such as – mammographic density [63], sociodemographic correlates [64], maternal age at last birth [65], employment and work schedule [66], endocrine disrupting chemicals and inflammation [10], childhood adversity and social support [67] and HIV positive and Negative pregnant women [68]. Data on parity was collected as a covariate and the statistical relationship between TL and parity was reported. 3. Studies that assessed pregnancy’s impact on TL and encompassed a longitudinal study design with multiple TL measurements throughout early pregnancy and early postpartum [10, 67–69]. Of these, only Zota et al. [10] included data on both parity and TL, assessing TL differences among nulliparous and multiparous women, and longitudinally throughout pregnancy, and we utilized both data sets for this review. In sum, we integrated data from all selected studies to enhance our understanding of both the telomere dynamics throughout pregnancy and the relationship between parity and women’s TL.

### Negative correlation

Of the 11 studies that assessed the relationship between parity and TL, four studies found a negative correlation; in general, having more children was correlated with shorter TLs. Kresovich et al. [58], who examined the relationship between reproductive history and TL via number of children and exposure to endogenous and exogenous estrogen has demonstrated an inverse correlation between TL and parity, and the correlation with parity was greatest for women with four or more births in comparison to women with 0 or 1 births. Interestingly, a positive relationship between breastfeeding duration and TL has been observed in this study [58]. Pollack et al. [60] who studied the association between parity and TL, found that parity was correlated, with shorter TLs compared to nulliparous women. Nonetheless, number of live births had an inverse U shape correlation with TL, showing a shorter T/S ratio for women with 1, 2 and ≥ 5 live births, demonstrating women with more than five live births showing the largest reduction in mean T/S ratio [60]. Ryan et al. [61], who studied the youngest age group within all studies in this review assessed gravidity in relation to TL and found that gravidity was inversely correlated with TL; each additional pregnancy was correlated with 0.34–3.67 years of telomere aging and this association was not related to SES [61]. Parks et al. [66] study assessing the relationship between work schedule and TL has shown that current full-time work was correlated with shorter TL, yet, this relationship was strongest for women with three or more children [66]. Of note, three of the four studies showing a negative correlation between parity and TL, based on the NOS checklist, received a high quality grade, demonstrating rigorous research methods [58, 60, 66].

### Positive correlation

One study found a positive correlation between TL and parity [57], showing that the more children the woman had, the longer her TL. This study demonstrated that each additional child born throughout the 13 year follow-up period was correlated with an 0.059 increase in TL units [57]. Based on the NOS checklist, this study was graded with a medium quality grade, due to its lack of methodological rigor questioning its results applicability to the general population of women.

### Non-correlation

Finally, six studies found no correlation between parity and TL. Of these, two studies assessed the relationship between TL and parity directly [59, 62] and four studies assessed the relationship between TL and a secondary variable, with parity assessed as a covariate [10, 63–65]. Four of these studies received a high-quality grade according to the NOS checklist [10, 59, 64, 65], and two received a medium quality grade [62, 63].

### Longitudinal correlation

Of the studies that assessed change in TL longitudinally throughout early pregnancy and early postpartum, three observed no change in TL over time [10, 67, 69] of these, two studies received a high quality grade according to the NOS checklist [10, 67] and one study received a medium quality grade [69]. At last, one study, who followed HIV positive and negative women throughout pregnancy, and received a high quality grade, has shown that both groups had similar TLs which increased throughout pregnancy, especially among women younger than 35 years [68].

### Mini meta-analysis results

**Fig. 2:**
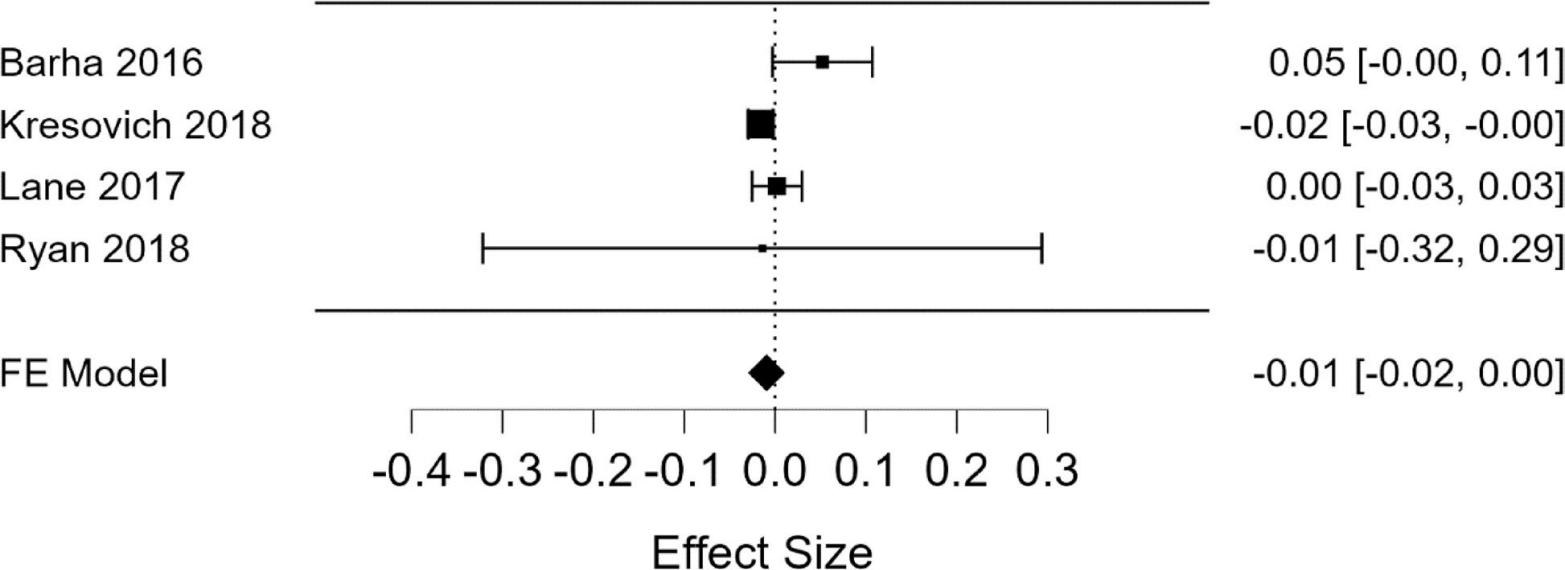
Forest Plot Mini Meta-Analysis* * Mini meta-analysis for the effect of parity on telomere length

The mini meta-analysis results obtained showed a non-significant estimated effect size of parity on TL. The Forest plot (Fig. 2) reveals a slight negative, yet, non-significant effect of parity on TL (ES = -0.009, *p* = 0.126, CI -0.021, 0.03).

## Discussion

The current systematic review sought to investigate recent research that assessed TL dynamics throughout pregnancy and the relationship between parity and women’s TL, using a systematic search strategy, we identified 14 relevant studies. Overall, out of 11 studies that examined parity and TL, four studies found a negative correlation between parity and TL, one study found a positive correlation, and six studies did not find any correlation. Of the four studies assessing telomere dynamics throughout pregnancy, three studies demonstrated no change in TL overtime and one study demonstrated telomere elongation from early pregnancy to postpartum.

Based on the quality assessment of the studies, the only study that has demonstrated a positive relationship between TL and parity [57] lacked methodological rigor, as it utilized a convenience sampling method and lacked controlling for key confounders. These deficiencies in methodology question the reliability of the positive relationship between parity and TL reported. Conversely, three of the four studies that demonstrated a negative correlation between parity and TL [58, 60, 66], scored highly for quality, exhibited relatively large sample sizes, controlled for various potential confounders and encompassed a wider range of ages. The six studies that demonstrated no correlation between parity and TL were a mixture of high and moderate quality studies and demonstrated heterogeneity in their study designs.

The mini meta-analysis results showed a negative trend in the association between parity and TL, although this trend was not statistically significant, the small number of studies included, limits the power of the meta-analysis, yet, warrants future research on the effect of parity on TL. Overall, these mixed study results may have arisen in part from the varied research methods utilized in each study for measurement of parity, timing of measures, and age of the women, as well as lack of statistical adjustment for potential confounders that possibly brought about these different study results. Our systematic review defined parity as the number of times a woman has given birth to a baby of viable gestation or fetal weight, regardless of the birth outcome. Nonetheless, parity is one aspect of reproduction, which is complex, as it is affected by many variables beyond parity, such as women’s’ nutritional and socioeconomic status (SES), breastfeeding status, childcare demands, social support, spacing and timing of pregnancies, as well as women’s’ reproductive lifespan including age at first and last reproduction [5].

Reproductive lifespan and estrogen exposure throughout, as well as women’s age at first and last pregnancy have been studied with regards to their effect on women’s’ mortality and TL. In terms of women’s’ age at pregnancy, Dior et al. [51] demonstrated a positive correlation between parity and mortality among women that were younger in age at first birth [51]. Concordantly, Parks et al. [66] demonstrated shorter TL for younger women at first birth, yet two other studies did not find a correlation between women’s age at first birth and TL [57, 63]. Interestingly, two studies have shown a positive relationship between maternal age at last birth and TL [65, 71], yet in one of these studies, this relationship was restricted to 1–2 children [65]. Increased parity might also imply a longer reproductive lifespan, as Lin et al. [35] demonstrated a positive correlation between reproductive lifespan, indicative of longer endogenous estrogen exposure (from menarche to menopause) and TL. Nonetheless, Kresovich et al. [58] has demonstrated that women’s longer reproductive periods, as well as increased parity were associated with shorter TL. Moreover, women with extended fertility, having children between 43–48 years of age exhibited longer telomeres in comparison to matched age fertile women who were unable to naturally conceive, yet this relationship was significant only for women with up to 8 children and non-significant for women with nine or more children [62]. The data presented may imply that the burden of childbirth is a contributary factor to women’s mortality and TL, yet, parity alone cannot represent reproduction, timing of pregnancies as well as women’s’ reproductive lifespan should be considered as well. Further studying the weighted contributions of different reproductive factors is essential to understand parity’s role in women’s longevity and TL.

Other stressors that may affect women’s TL include women’s own fetal stressful environmental exposures [72], adverse childhood experiences [73], psychological and psychosocial stressors [74, 75], long term exposure to environmental stressors [10], chronic disease [8] and lifestyle behaviors such as diet [18, 21], physical activity [20] and sleeping patterns [19]. Nonetheless, many studies do not collect these measures and incorporate them in their statistical analysis.

Interestingly, four of the six studies in this review, which found no relationship between parity and TL (all graded with high quality scores according to the NOS checklist), included mainly premenopausal younger women aged 38–45 [59], 18–42 [10], 43–48 [62] and mean age 37 ± 6 [64], Whereas, two of the four studies in this review that had a broader range of ages including pre and post-menopausal women, showed a negative correlation between parity and TL, these studies also ranked highly according to NOS checklist [58, 66]. Age may be an important factor in this relationship. Telomere dynamics vary throughout the life span and TL shortening may occur in a nonlinear fashion  [76–78]. So far, evidence points to enhanced telomere attrition early in life (birth to 4 years), followed by periods of maintenance mostly observed during the first and third decades, and gradual TL shortening thereafter [76]. Although more research is needed to ascertain telomere dynamics throughout the life span, this variable might explain the lack of relationship between parity and TL among many of the studies with younger age groups. In addition, studying younger women may assess the short-term effects of parity on TL, making it difficult to determine the long-term consequences of repeated pregnancies on TL.

Different definitions of parity in each study make it difficult to compare results across studies. Comparing studies counting live births with studies including miscarriages and stillbirths within the definition of parity may be biased as telomere shortening may be influenced by stress endured via these events rather than by the biological occurrence of childbirth. Ryan et al. [61] found a negative correlation between parity and TL, yet parity in this study also included pregnancies that ended up as stillbirths and miscarriages, which diverges from our definition of parity and possibly affecting the direction and strength of the relationship. Lane Cordova et al. [59], who defined parity as any pregnancy lasting more than 20 weeks, possibly resulting in an infant with a lower survival rate, has shown no correlation between parity and TL. Noteworthy, two studies that defined parity as a binary variable of either nulliparity or multiparity have shown no correlation between parity and TL [10, 63]. Therefore, standardizing a definition of parity is crucial for accurate study conclusions and comparison of results across studies.

Beyond the various definitions of parity, we explored the categories of parity defined in each study, and examined whether the relationship between parity and TL is possibly non-linear. Within the studies that assessed parity as a primary measurement (*n* = 6), and the studies that assessed parity as a covariate (*n* = 5), only five and three studies in each group respectively, categorized parity in a way that we could attempt to infer whether a certain number of children seems to be the threshold for telomere changes**.** Of these, one primary study has found a positive correlation between parity and TL [57], yet two primary studies [59, 62] and two studies that assessed parity in relation to TL as a covariate [64, 65] did not find a correlation between parity and TL. Nonetheless, based on two studies that assessed the relationship between parity and TL directly [58, 60] and one study that utilized parity as a covariate [66] and based on their relatively large sample sizes *n* = 1048 [58], *n* = 1554 [60] *n* = 608 [66] and their high quality score ratings according to the NOS, this potential nonlinear relationship should be further explored, yet cannot be concluded from the data presented in these studies. Beyond the biological effect of parity on TL, a social factor might be considered as lower SES is associated with higher parity, poorer health, and shorter TL [79, 80]. A lower SES may signify less available resources that may lower energy requirements for childrearing. Moreover, higher parous women might endure more stress from raising more children, which is associated with poorer health outcomes [81], yet, conversely, have a broader social network, therefore, more social support, which is linked to better health and longer TLs [82]. Barha et al. [57] who demonstrated TL was longer in multiparous women highlights the role of social support in health status, aging, and TL. Generally, social relationships are associated with enhanced physical and emotional health outcomes and a lower incidence of mortality among individuals [83] evidenced by research investigating the association between social ties and TL- having social ties is associated with longer telomeres [84, 85]. A positive social environment may contribute to women’s’ health and negate the challenging and stressful postpartum period demands [86]. Barha et al. [57] attributed the positive relationship between parity and TL, to the cooperative breeding strategy, characterized by the Mayan population studied. Feasibly, having more children increase social support received, which in turn, may lower energetic costs of childrearing, leaving more energy for maintenance therefore, slowing biological aging [57]. Thus, the effect of social support on TL among women in the general population requires further investigation.

## Conclusions

Reproduction is a complex biological mechanism, taxing women’s energy demands, increasing their susceptibility to specific chronic disease, yet decreasing it for others. Overall, pregnancy can potentially enhance biological aging, yet also provide women with biological health-protective pathways. Adding to the complex nature of this phenomenon, additional variables such as childrearing, stress, social support, and lifestyle factors may affect TL and should be considered when researching the effects of reproduction on TL. Our review suggests that pregnancy does not affect TL throughout the pregnancy period. The studies in this review focusing on pregnancy, utilized a longitudinal research design, measuring TL during early and late pregnancy or early postpartum. Three of four studies reviewed showed no significant change in TL throughout pregnancy [10, 67, 69] and the one study that has demonstrated elongated telomeres throughout pregnancy has studied women with HIV [68]. Even though the healthy control group also demonstrated elongated telomeres, their small sample size (*n* = 41), necessitates further research.

Overall, three of the four studies demonstrating a negative correlation between parity and TL demonstrated rigorous research methods, these can possibly imply that having more children may be associated with enhanced telomere attrition. The six studies that exhibited no correlation between parity and TL, although most were high quality studies according to the NOS, most of these studies included narrower younger age ranges, questioning whether the burden of childbirth effecting TL manifests later in life. Noteworthy, the one study that found a positive correlation between parity and TL, besides exhibiting flaws in its methodology, studied a unique population—Kaqchikel Mayan women in Guatemala [57] that do not necessarily represent the general population of women. At last, the mini meta-analysis did not find a statistically significant effect of parity on TL, although a negative trend was observed. Future meta-analyses with a larger number of studies may provide more conclusive evidence on this topic.

Further research is needed to better understand the dynamics of pregnancy on TL and the effects of parity on TL over time. For more accurate conclusions, clearly defining parity is crucial, alongside comparing different age groups of pre- and post-menopausal women and statistically adjusting for the potential confounders during pregnancy and during child raising years. Additionally, measuring telomerase, might clarify the pathway involved in the pregnancy, parity and TL association, since estrogen elevated during pregnancy, has been associated with increased telomerase activity, which in turn promotes telomere elongation [34, 87].

There are limitations to the studies reviewed. Several of the studies had sample sizes under 100, and it is hard to find cross sectional effects of parity given the already high individual variance in TL between people. Longitudinal studies across one or multiple pregnancies would offer the best design. Moreover, TL measurements can vary depending on the testing techniques used and consequently affect the association between parity and TL, therefore the lack of standardized TL measurement techniques across studies may limit the comparability of findings. Future research should strive for consistency in TL measurement protocols to enhance the reliability and validity of associations with other variables, including parity. In addition, almost all studies in this review used the PCR method that may not have been able to detect small changes during short periods that fall below the threshold of measurement error. Of note, qPCR measurements can be susceptible to measurement error, because it’s a complex technique that can be affected by a number of factors, including the quality of the DNA sample, the concentration of primers and probes utilized throughout testing, and the settings of the PCR machine. All these can affect the accuracy of TL measurements [88]. Furthermore, there is considerable variability observed both within individual samples and across different samples when employing the qPCR method for TL assessment [89]. Further studies are thus needed using the more precise southern blot measurement method) TRN Recommendations for the measurement of telomere length in population studies (Telomere research Network [TRN]), n.d.) [90].

### Supplementary Information

**Additional file 1: Appendix 1.** Full Search Strategy. **Appendix 2.** General Keyword Search Strategy.

## Data Availability

All data generated or analysed during this study are included in this published article [and its supplementary information files].

## Abbreviations

TL: Telomere Length
NOS: Newcastle–Ottawa scale
