# Supplementary material for: Are pregnancy and parity associated with telomere length? A systematic review
Source: BMC Pregnancy Childbirth. 2023 Oct 17;23:733. doi: 10.1186/s12884-023-06011-8 (PMC10583451; doi:10.1186/s12884-023-06011-8)
Supplement: Supplementary file 1 — Additional file 1: Appendix 1. Full Search Strategy. Appendix 2. General Keyword Search Strategy. [file 12884_2023_6011_MOESM1_ESM.pdf]

Appendix 1: Full Search Strategy

General search terms used in all	Telomere* OR telomerase **AND** length* OR short* OR long* OR elongat* OR homeostasis OR maintenance OR measur* OR attrition OR dynamics **AND** Obstetric* OR Premenopaus* OR Gestation* OR Gravidit* OR Nulligravidit* OR Primigravidit* OR Multigravidit* OR Parity OR Nulliparous OR Primiparity OR Multiparity OR Nulliparity OR Pregnan* OR Mother***** OR maternal OR Prenatal OR antenatal OR Perinatal OR Postnatal OR Childbirth OR birth OR delivery OR labor OR labour OR Conception OR Preconception OR Parental OR postpartum OR reproducti* OR parturition OR lactat* OR " breast feed*" OR breastfeed* OR "breast fed" OR breastfed
Pubmed/Cochrane Mesh terms	Telomere (MeSH) OR Telomerase (MeSH) OR Telomere Shortening (MeSH) OR Telomere Homeostasis (MeSH) **AND** Delivery, Obstetric (MeSH) OR Labor, Obstetric (MesH) OR Premenopause (MeSH) OR Live Birth (MeSH) OR Term Birth (MeSH) OR Reproductive History (MeSH exp) Pregnancy (MesH) OR Parturition (MeSH) OR Natural Childbirth (MeSH) OR Pregnant Women (MeSH) OR Postpartum Period (MeSH) OR Maternal-Fetal Relations (MeSH) OR Mothers (MeSH) OR Maternal-Fetal Exchange (MesH) OR Reproduction (MeSH) OR Reproductive Behavior (MeSH) OR Reproductive Physiological Phenomena (MeSH) OR Maternal age (MeSH) OR Lactation (MeSH) OR Breast feeding (MeSH)
Psycinfo Thesaurus of Psychological Index Terms	Telomeres (IT) **AND** Obstetrics (IT) OR Pregnancy Outcomes (IT) OR Pregnancy (IT) OR Sexual Reproduction (IT) OR birth (IT) OR Reproductive Health (IT) OR Primipara (IT) OR Perinatal Period (IT) OR Postnatal Period (IT) OR Mothers (IT) OR Natural Childbirth (IT) OR Expectant Mothers (IT) OR Prenatal Care (IT) Parental Investment (IT) OR Lactation (IT) OR Breast feeding (IT)
Cinahl Subjest Headings	Telomere (IT) OR Telomerase (IT) **AND** Delivery, Obstetric (IT) OR Intrapartum Care (IT) OR Perinatal Care (IT) OR Postnatal Care (IT) OR Prenatal Care (IT) OR Premenopause (IT) OR Reproduction (IT) OR sexual reproduction periods (IT) OR Periconceptual Period (IT) OR Postnatal Period (IT) OR Pregnancy (IT) OR Parity (IT) OR Nulliparas (IT) OR Multiparas (IT) OR Expectant Mothers (IT) OR Mothers (IT) OR Parents (IT) OR Childbirth (IT) OR Labor (IT) OR Maternal-fetal exchange (IT) OR Mothers- infant relations (IT) OR Primiparas (IT) OR Maternal behavior (IT) OR Maternal role (IT) OR Prepregnancy care (IT) OR Obstetric care (IT) OR Lactation (IT) OR Breastfeeding (IT) OR Term birth (IT) OR Obstetrics (IT) OR Maternal age

Appendix 2: General Keyword Search Strategy

Concept #1- Telomere terms	Concept #2 - Length terms	Concept #3- Parity terms
Telomere*<br>telomerase	length*<br>short*<br>long*<br>elongat*<br>homeostasis<br>maintenance<br>measur*<br>attrition<br>dynamics	Obstetric*<br>Premenopaus*<br>Gestation*<br>Gravidit*<br>Nulligravidit*<br>Primigravidit*<br>Multigravidit*<br>Parity<br>Nulliparous<br>Primiparity<br>Multiparity<br>Nulliparity<br>Pregnan*<br>Mother*<br>maternal<br>Prenatal<br>antenatal<br>Perinatal<br>Postnatal<br>Childbirth birth<br>delivery<br>labor<br>labour<br>Conception<br>Preconception<br>Parental<br>postpartum<br>reproducti*<br>parturition<br>lactat*<br>" breast feed*"<br>breastfeed*<br>"breast fed"<br>breastfed
